# High-fidelity optical fiber microphone based on graphene oxide and Au nanocoating

**DOI:** 10.1515/nanoph-2023-0209

**Published:** 2023-09-20

**Authors:** Liangtao Hou, Yan Li, Libin Sun, Chao Liu, Yichao Zheng, Yi Liu, Shiliang Qu

**Affiliations:** Department of Optoelectronics Science, Harbin Institute of Technology, Weihai 264209, China; Sino-German Joint Research Center of Advanced Materials, School of Materials Science and Engineering, East China University of Science and Technology, Shanghai 200237, China; School of Physics, Harbin Institute of Technology, Harbin 150001, China

**Keywords:** optical fiber microphone, graphene oxide, Au nanocoating, high-fidelity, real-time communication

## Abstract

A high-fidelity optical fiber microphone (HF-OFM) with hybrid frequency and fast response is theoretically and experimentally demonstrated by the nanofabrication techniques for real-time communication, which consists of a graphene oxide (GO) film, an Au nanocoating, and an air cavity. The internal stress of the film is increased by the method of mechanical tensile preparation, and the microphone response flatness is improved. Meanwhile, the structural design of the 3 nm Au nanocoating improves the acoustic pressure detection sensitivity by 2.5 times by increasing the reflectivity. The experimental result shows that single, dual, and triple frequency acoustic signal detection in the frequency range of 0.1 kHz–20 kHz are achieved with acoustic pressure sensitivities of 9.64, 9.66, and 8.9 V/Pa, as well as flat frequency response (<2 dB variation). The minimum detectable pressure (MDP) at 1 kHz is 63.25 μPa/Hz^1/2^. In addition, the high-fidelity real-time transmission of audio signals over an angle range of −90° to 90° is verified by a self-made acoustic pressure detection device. Such a compact, high sensitivity, and large measurement range HF-OFM is very promising for applications of oil leakage exploration, acoustic source location, and real-time communication.

## 1 Introduction

Acoustic detection plays an important role in various fields, ranging from household noise meters to national defense security protection [1, 2]. Compared with traditional electrical acoustic sensors [3], optical fiber acoustic sensors have attracted much attention in the fields of photoacoustic gas measurement [4], photoacoustic imaging [5], pipeline leakage [6], and natural disaster early warning [7] due to their advantages such as low cost, small size, corrosion resistance, high sensitivity, and antielectromagnetic interference. Currently, optical fiber acoustic sensors of various principles have been reported, such as Mach–Zehnder interferometer [8], Michelson interferometer [9], Sagnac interferometer [10], and long-period fiber grating [11]. Due to the low sensitivity, this type of sensor usually requires a membrane transducer to assist in the detection of acoustic signals, which increases the size of the sensor. Therefore, with the advantages of high sensitivity and compact structure, Fabry–Perot interferometer (FPI) has gradually become a research hotspot.

In recent years, a variety of film-based FPI acoustic sensors have been proposed, and their sensitivity and response range mainly depend on the physical properties of the film such as thickness, diameter, Young’s modulus and Poisson’s ratio, etc. There are many kinds of materials used for the vibration film of FPI acoustic sensor, mainly divided into metal films based on Au [12], silver [13], and aluminum [14]; polymer films based on polydimethylsiloxane [15] and chitosan [16]; and two-dimensional material films based on graphene oxide [17] and molybdenum disulfide [18]. For metal and polymer films, the sensitivities are usually increased by enlarging the film diameter or reducing the film thickness, but the flat working range of frequency will also be affected. Therefore, FPI acoustic sensors based on wheel-like structure [19], optomechanical microresonator [20], cantilever beam [21], and microbubble [22] have been reported to solve the problem of low sensitivity. However, femtosecond laser processing and pump light heating are required, which greatly increases the cost and difficulty of preparation. Graphene oxide (GO) is a promising vibrating membrane material due to its nanometer thickness, high mechanical elasticity, wide flat response range, and excellent biocompatibility with silicon [23]. At present, the methods for growing GO film are mainly chemical vapor deposition [24] and physical vapor deposition [25, 26], both of which require chemical corrosion and film secondary transfer, and the flatness and uniformity of the films in the secondary transfer process need to be carefully controlled. Especially in order to realize the detection of acoustic signal, the method of changing the geometric size of the film is usually used. Although the detection sensitivity is improved, the response flatness is not guaranteed [27]. Especially in the multi-frequency environment such as speech recognition, the flat response is greater than 10 dB in the frequency range of 0–20 kHz [27, 28]. Therefore, how to improve the response flatness while ensuring the detection accuracy is an urgent challenge to be solved.

In this paper, we proposed a high-fidelity optical fiber microphone (HF-OFM) based on multi-frequency acoustic measurements by the nanofabrication techniques, consisting of a GO film, an Au nanocoating, and an air cavity. Using numerical and finite element analysis methods, the pressure sensitivity of the 718 nm thickness and 308 µm diameter GO film is −6.89 nm/kPa with the resonant frequency of 315.87 kHz. Through mechanical stretching method, the 3 nm Au nanocoating and 58.95 µm air cavity, the signal-to-noise ratio (SNR), and the minimum detectable pressure (MDP) are further effectively increased to 53.05 dB and 63.25 μPa/Hz^1/2^ at 1 kHz with the acoustic pressure sensitivity of 9.64 V/Pa, as well as flat frequency response (<2 dB variation). Based on this, combined with the wide bandwidth of the GO film, dual-frequency and triple-frequency acoustic signals were tested with sensitivities of 9.66 V/Pa and 8.9 V/Pa and SNRs of 50.01 dB/49.79 dB at 1/1.1 kHz and 48.13/47.24/47.73 dB at 1/1.1/1.2 kHz, respectively. In addition, the amplitude of the acoustic signal decreases by less than 1/3 over an angle range of −90° to 90°. The microphone achieves high fidelity transmission of audio signals with flat response and high acoustic pressure sensitivity at multiple frequencies. With low-cost, high stability, and compact, the proposed HF-OFM is very promising for the applications in oil leakage exploration, acoustic source location.

## 2 Principles and simulations

### 2.1 Working principle

**Figure 1:**
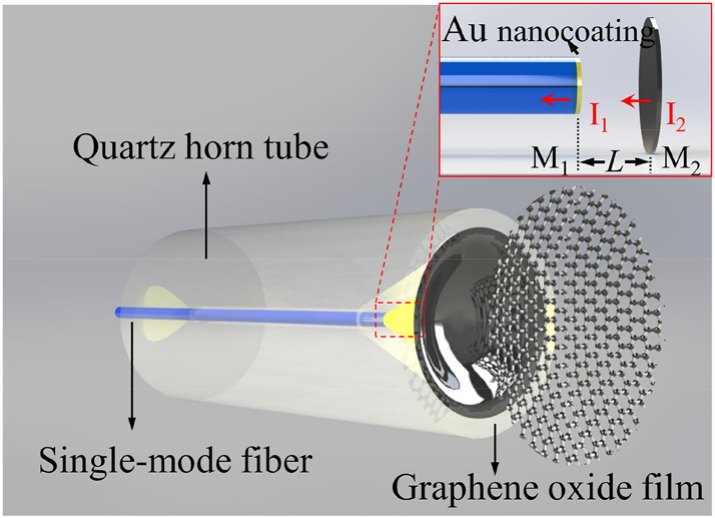
Schematic diagram of the HF-OFM.

The schematic diagram of the proposed HF-OFM is shown in Figure 1(a), which consists of single-mode fiber (SMF), quartz horn tube, Au nanocoating, GO film, and air cavity. Due to the existence of the refractive index (RI) difference, Fresnel reflection occurs at the Au–air surface and air–GO surface that defined as M_1_ and M_2_. Meanwhile, the thickness of the GO film is on the order of nm, and the structure can be approximated as two reflected light beams from M_1_ and M_2_ are coupled back to the SMF core to form Fabry–Perot interference.

According to the two-beam interference theory, the interference light intensity could be expressed as [29]

(1) $$I=I_{1}+I_{2}+2\sqrt{I_{1}I_{2}}\cos\left(\frac{4\pi nL}{\lambda }+\varphi_{0}\right)$$

where *I*_1_ and *I*_2_ are the light intensities of the reflected light behind M_1_ and M_2_, respectively. The intensity of the reflected light is only related to the wavelength, and *I*_1_ and *I*_2_ can be approximated as constants when the wavelength is fixed. Assuming *n* is the RI of air, *L* is the length of air cavity, *λ* is the input wavelength, and *φ*_0_ is the initial phase.

When the external acoustic pressure is applied to the GO film, the deformation of the GO film causes the FP cavity to decrease. Whereafter, the change of interference light intensity can be written as

(2) $$\Delta I=-\frac{8\pi }{\lambda }\sqrt{I_{1}I_{2}}\sin\left(\frac{4\pi nL}{\lambda }\right)\Delta L$$

where Δ*L* is the change of *L*. When the amplitude of GO film vibration caused by the applied acoustic pressure is less than 30 % of its thickness, there is a good linear relationship between GO film deformation and acoustic pressure. In view of the boundary of the GO film is fixed at the quartz horn tube, the deformation at the center of the film when the total input power of the external acoustic signal is constant can be expressed as [27, 30]

(3) $$\Delta L=\underset{n}{\sum }\frac{3\Delta PR^{4}\left(1-\mu^{2}\right)}{16Et^{3}}\frac{{f_{m}}^{2}}{\sqrt{{\left({f_{m}}^{2}-{f_{n}}^{2}\right)}^{2}+4{f_{n}}^{2}\xi^{2}}}$$

where Δ*P* is the change of acoustic pressure. *R*, *μ*, *E*, and *t*, respectively, represents the radius, Poisson’s ratio, Young’s modulus, and thickness. *f*_*n*_ is the frequency of the acoustic wave, and *ξ* is the damping coefficient. *f*_*m*_ is the *m*th-order resonant frequency of the GO film, which can be described as

(4) $$f_{m}=\frac{{k_{m}}^{2}t}{4\pi R^{2}}\sqrt{\frac{E}{3\rho \left(1-\mu^{2}\right)}}$$

where *k*_*m*_ is the *m*th-order constant coefficient, and *ρ* is the mass density of the GO film. From Eq. (4), the resonant frequency is only related to the physical properties of the GO film. According to Eq. (3), the acoustic pressure sensitivity can be expressed as

(5) $$S=\frac{\Delta L}{\Delta P}=\underset{n}{\sum }\frac{3R^{4}\left(1-\mu^{2}\right)}{16Et^{3}}\frac{{f_{m}}^{2}}{\sqrt{{\left({f_{m}}^{2}-{f_{n}}^{2}\right)}^{2}+4{f_{n}}^{2}\xi^{2}}}$$

According to Eq. (5), it can be found that the thickness and radius of the GO film have a crucial effect on the sensitivity of the HF-OFM. The periodic variation of the acoustic signal frequency can be demodulated by detecting the amplitude variation of the light intensity. Meanwhile, the closer the input frequency is to the resonant frequency, the worse the flat response of the microphone. Further, the finite element analysis method is used to further verify the mechanical characteristics and frequency response characteristics of the HF-OFM at various thicknesses and radii.

### 2.2 Sensor design

**Figure 2:**
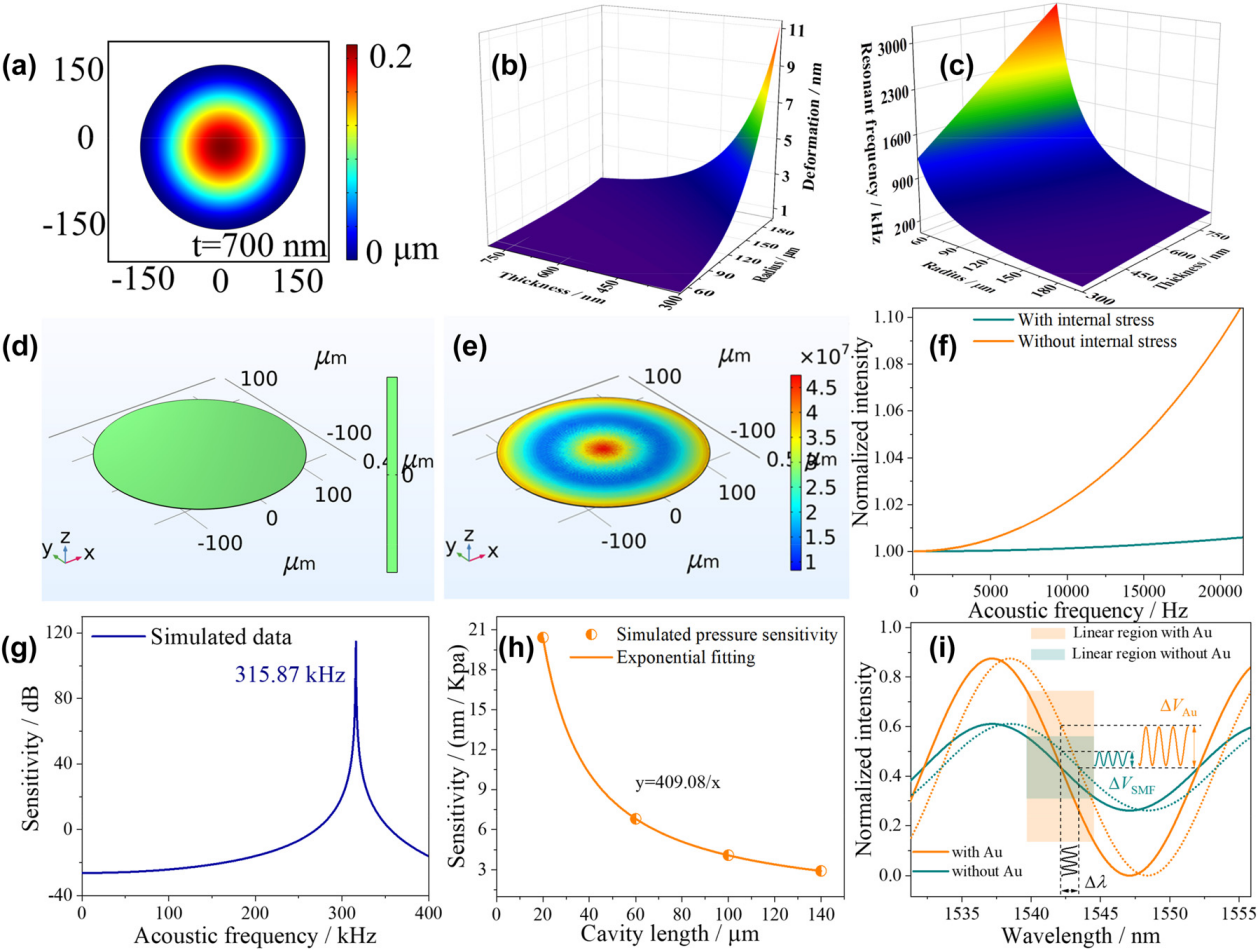
Simulation optimization of HF-OFM structural parameters using finite element analysis and numerical simulation analysis. (a) Deformation amplitude of GO film. (b) Deformation and (c) resonant frequency under different radii and thickness. Force analysis of GO films prepared by (d) physical vapor deposition and (e) mechanical stretching method. (f) Sensitivity responses of the film with and without internal stress at different acoustic frequencies. (g) Acoustic pressure sensitivities under different acoustic frequencies. (h) The simulated relationship between pressure sensitivity and *L*. (i) The simulated intensity response at different extinction ratios.

Through the finite element analysis method, the 3-D model of GO film with diameters of 300 μm was constructed. In terms of GO film, the *μ*, *E*, *ρ*, and *t* of GO film are 0.17, 1 Tpa, 2.2 × 10^3^ kg/m^3^, and 700 nm, respectively [29]. When the external pressure acts on the GO, the film deformation will occur, and the deformation value at the center point is the largest, as shown in Figure 2(a). Figure 2(b) shows the simulation of the relationship between the deformation at the center of the GO film and radius, thickness of the GO film at 1 Pa. The results show that the deformation of the center point increases with the decrease of the thickness and the increase of the radius, that is, the sensitivity of the HF-OFM increases. Through calculation, the sensitivity is proportional to the fourth power of the increase value of the GO film radius and inversely proportional to the third power of the increase value of the thickness. While the increased surface area of the film helps to improve sensitivity, the roughness of the film introduces more noise and limits the flat frequency operating range of the microphone. As shown in Figure 2(c), the resonant frequency decreases with increasing radius and decreasing thickness, and the results show that the resonant frequency are proportional to the thickness and inversely proportional to the square of the radius. Meanwhile, the decrease of film thickness will affect the mechanical strength, which limits the application of the HF-OFM in harsh environments. Therefore, after comprehensive consideration, the radius of the film is 150 μm and the thickness of the film is 700 nm to improve the acoustic pressure sensitivity. On this basis, the mechanical stretching method is used to optimize the flat response characteristics.

In order to further study the influence of the internal stress of the film on the performance of the microphone, finite element analysis method was used to simulate the stress of the film prepared by physical vapor deposition method, as shown in Figure 2(d). The prepared GO film is directly adsorbed on the end face of hollow quartz tube, and the surface of the film will not be affected by internal stress. In contrast, the GO film prepared by mechanical stretching method is subject to the action of surface tension during the preparation process, and the internal stress on the surface of the film can reach 45 MPa, as shown in Figure 2(e). Figure 2(f) shows the sensitivities response of the film with and without internal stress at different acoustic frequencies. Compared with the traditional physical vapor deposition method, the response flatness of the microphone prepared by the mechanical stretching method is improved by 16.5 times in the frequency range of 0–20 kHz. The corresponding resonant frequency is shown in Figure 2(g), which is 315.87 kHz. In the flat range of 0 Hz to 20 kHz, the sensitivity fluctuation is only ∼1 dB, which can realize real-time transmission and detection of acoustic signals with high fidelity.

The gas pressure sensitivity will directly determine the detection limit of acoustic pressure. The proposed optical fiber microphone is a Fabry–Perot interferometer, and the length of the interference cavity affects the wavelength sensitivity of the structure, which is related to the acoustic pressure response. Through numerical simulation analysis, the relationship between the pressure sensitivity of the structure and cavity length *L* is shown in Figure 2(h). The sensitivity increases exponentially as the cavity length decreases, and the relation is *y* = 409.08/*x*. Therefore, appropriate reduction of *L* can effectively improve the limit of acoustic pressure detection. Due to the limitation of tunable laser wavelength range, the corresponding interference spectrum with short cavity length is difficult to show a complete cycle in the wavelength range of 1520–1560 nm, which in turn affects the selection of *Q* point. The structure preparation and response characteristics of the microphone are comprehensively considered, and the initial cavity length is selected as 60 μm.

The extinction ratio (ER) of HF-OFM also plays an important role in improving the acoustic pressure sensitivity. According to the above theoretical optimization results, the initial cavity length, diameter, and thickness of the GO film are set as 60 μm, 300 μm, and 700 nm, respectively. Assuming the RIs of Au film, GO, and air are 0.52, 3.5, and 1, respectively. According to Eq. (1), the reflection spectra corresponding to coated/uncoated Au can be simulated by numerical calculation method, as shown in Figure 2(i). When the intensities of the two reflected lights are equal, the ER of the reflection spectrum has a maximum value. According to the Fresnel formula, the Au nanocoating has a larger RI difference with the SMF core than air. Therefore, the reflection spectrum ER with Au nanocoating is significantly increased by 0.525. In order to obtain the maximum acoustic pressure sensitivity, the central wavelength of the tunable narrow-bandwidth laser should be set at *Q* point, which is the maximum slope of the reflection spectrum curve, in the subsequent acoustic frequency test. In the linear region near *Q* point, the amplitude of the output signal after the acoustic signal modulates the optical wavelength is the largest and has no distortion. Applying the same pressure to the reflection spectra with and without Au nanocoating, it is found that the wavelength change Δ*λ* at the center point are all 1.31 nm. On the contrary, the change of spectrum intensity of Au nanocoating Δ*V*_Au_ is obviously increased by 0.1 compared with Δ*V*_SMF_. Meanwhile, the wavelength range of the linear region is all 4.83 nm. Therefore, Au nanocoating can effectively improve the acoustic pressure sensitivity of the HF-OFM.

### 2.3 Sensor preparation

**Figure 3:**
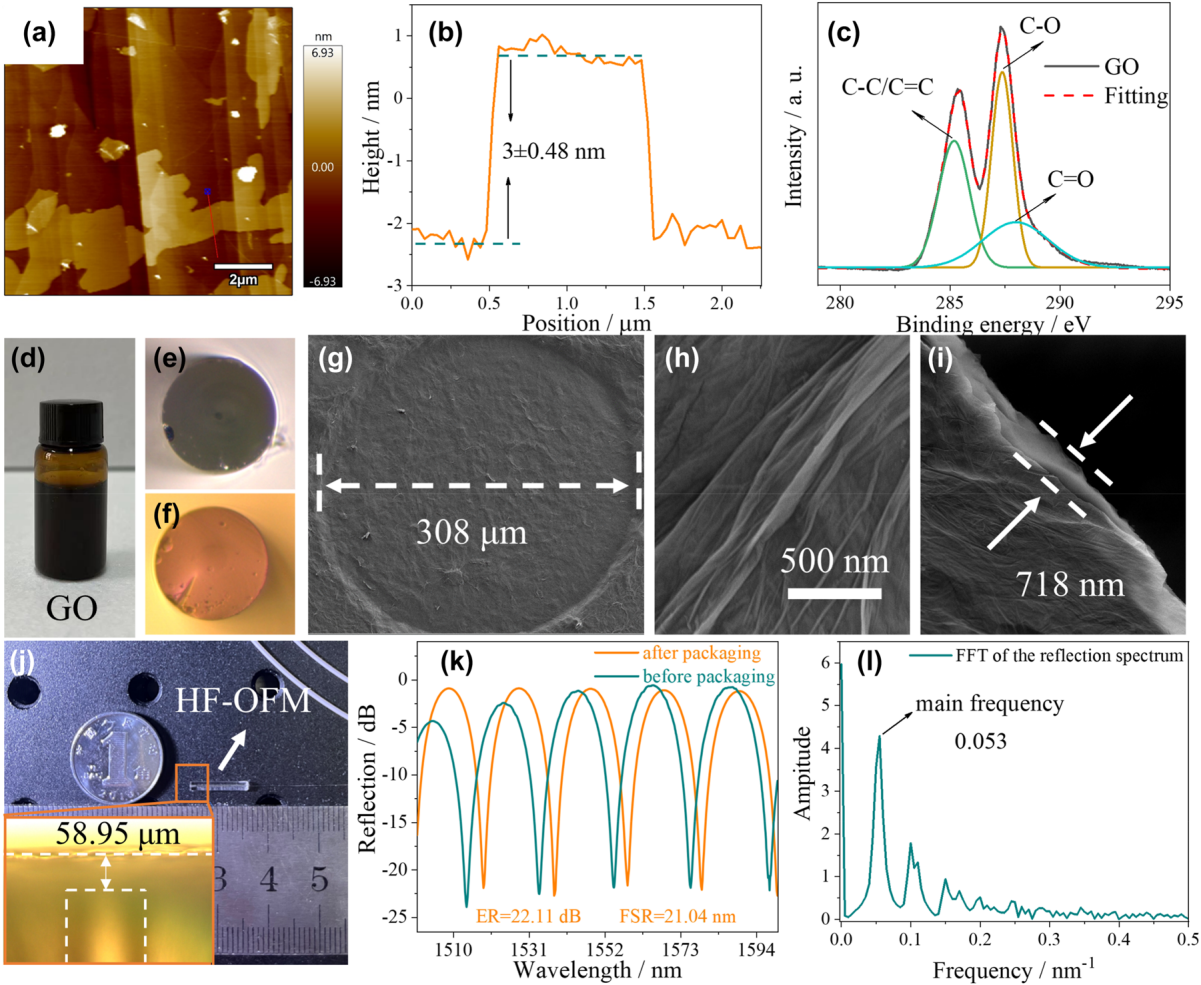
Structural characterization of HF-OFM and corresponding spectral analysis. (a) Atomic force microscopy (AFM) image of the morphology of the GO. (b) The height profile of GO. (c) X-ray photoelectron spectroscopy (XPS) patterns of the GO. (d) Picture of the GO solution. SMF cross-sectional image (e) without and (f) with Au nanocoating. (g) and (h) Scanning electron microscope (SEM) image of the HF-OFM cross section and (i) thickness calibration of GO film. (j) Picture of the proposed HF-OFM, and (k) corresponding reflection spectrum and (l) frequency-domain spectrum.

The picture of GO solution with a concentration of 1 mg/mL is shown in Figure 3(d). The surface of the GO film exhibits a continuous morphology as observed from the atomic force microscopy (AFM) image shown in Figure 3(a). We have further characterized the thickness of the GO film, and the thickness estimated from Figure 3(b) is ∼3 nm, which is about the thickness of four monolayer GO deposits [31]. The composition mechanism of GO was further reveled by X-ray photoelectron spectroscopy (XPS) analysis. Figure 3(c) shows the three primary chemical environments of GO, corresponding to C–C/C=C, C–O, and C=O bonding at binding energies of 285.2, 287.2, and 287.8 eV, respectively [32]. The introduction of oxygen-containing groups not only makes GO chemically stable but also has good biological adaptability in the process of binding with optical fiber.

The preparation methods of HF-OFM are mainly divided into three nanofabrication technologies: gold coating, GO film deposition, and FP cavity assembly. Firstly, 10 mL GO solution with a concentration of 5 mg/mL was melted into 40 mL deionized water with a pipette and then fully mixed in the ultrasonic cleaner for 20 min to get a uniform GO solution with a concentration of 1 mg/mL. Next, the quartz horn tube with the horn shaped end of 308 μm diameter was slowly vertically lowered to contact with the GO solution. Here, the horn shaped end of the quartz horn tube in order to increase the diameter of the GO film to improve the acoustic pressure sensitivity. After 2 s, the quartz horn tube with the GO solution was quickly lifted due to the surface tension. The structure was placed in a drying box with a temperature of 60 °C for 1 h, and the uniform GO film could be firmly adsorbed on the end face of the quartz horn tube by van der Waals force. The SEM images of the prepared GO film are shown in Figure 3(g)–(i), with the diameter of 308 μm and the thickness of 718 nm. A section of SMF (Corning SMF-28) with a core/cladding diameter of 8.3/125 µm was then cut flat on the end surface and cleaned by arc discharge. According to our previous work [29], the Au nanocoating with ∼3 nm thickness was deposited on the end surface of SMF by sputter coater (Cressington, 108 Auto). Figure 3(e) and (f) shows the cross-sectional microscopic images of the SMF tip without and with Au nanocoating. Finally, the SMF tip with Au nanocoating was inserted into the quartz horn tube on a 3-D displacement platform to form the FP cavity. The process was observed by charge-coupled device (CCD) and optical spectrum analyzer (OSA, YOKOGAWA, AQ6370B, with the resolution of 0.02 nm) in real time. When the observed reflection spectrum has a free spectrum range (FSR) of 18.75 nm at 1500 nm, the SMF and the quartz horn tube are fixed with UV curable adhesive (SU-8, GM1070). In order to avoid contamination of the fiber end face with UV curable adhesive and maintain a stable *Q* point, the UV curable adhesive is cured as soon as it spills from the horn shaped side. According to FSR = *λ* ^2^/2*nL*, the distance between SMF end face and GO film is 60 μm, which is consistent with the value of the actual prepared cavity length in subfigure of Figure 3(j). Figure 3(j) shows the fabricated HF-OFM with the size of 1.8 mm × 11 mm (diameter × length), which proves that the proposed sensor has a good application prospect in the narrow and complex environment.

Thus, an FPI was formed with a cavity length of 58.95 µm, which was demodulated through the reflection spectrum measured by using the optical spectrum analyzer and broadband light source (BBS, with the range of 1200–1700 nm) connected by a circulator. The results of the comparison of the reflection spectra before and after packaging of the FPI structure are shown in Figure 3(k). The FSR and ER of the packed interference spectrum change slightly. From Figure 3(k), the ER and FSR of the HF-OFM are 22.11 dB and 21.04 nm, respectively. Compared with Figure S1(a), the Au nanocoating can effectively improve the ER of the reflection spectrum by 9.62 dB. Moreover, the ER fluctuation in the range Au nanocoating thickness of 3–15 nm is only ±0.65 dB, as shown in Figure S1(b). The reflection spectrum with Au nanocoating is transformed by fast Fourier transform (FFT), and the corresponding frequency-domain spectrum is shown in Figure 3(l). There is only a main frequency of 0.053 in the figure, which proves that the sensor is formed by dual beam interference.

## 3 Experiment results and discussions

**Figure 4:**
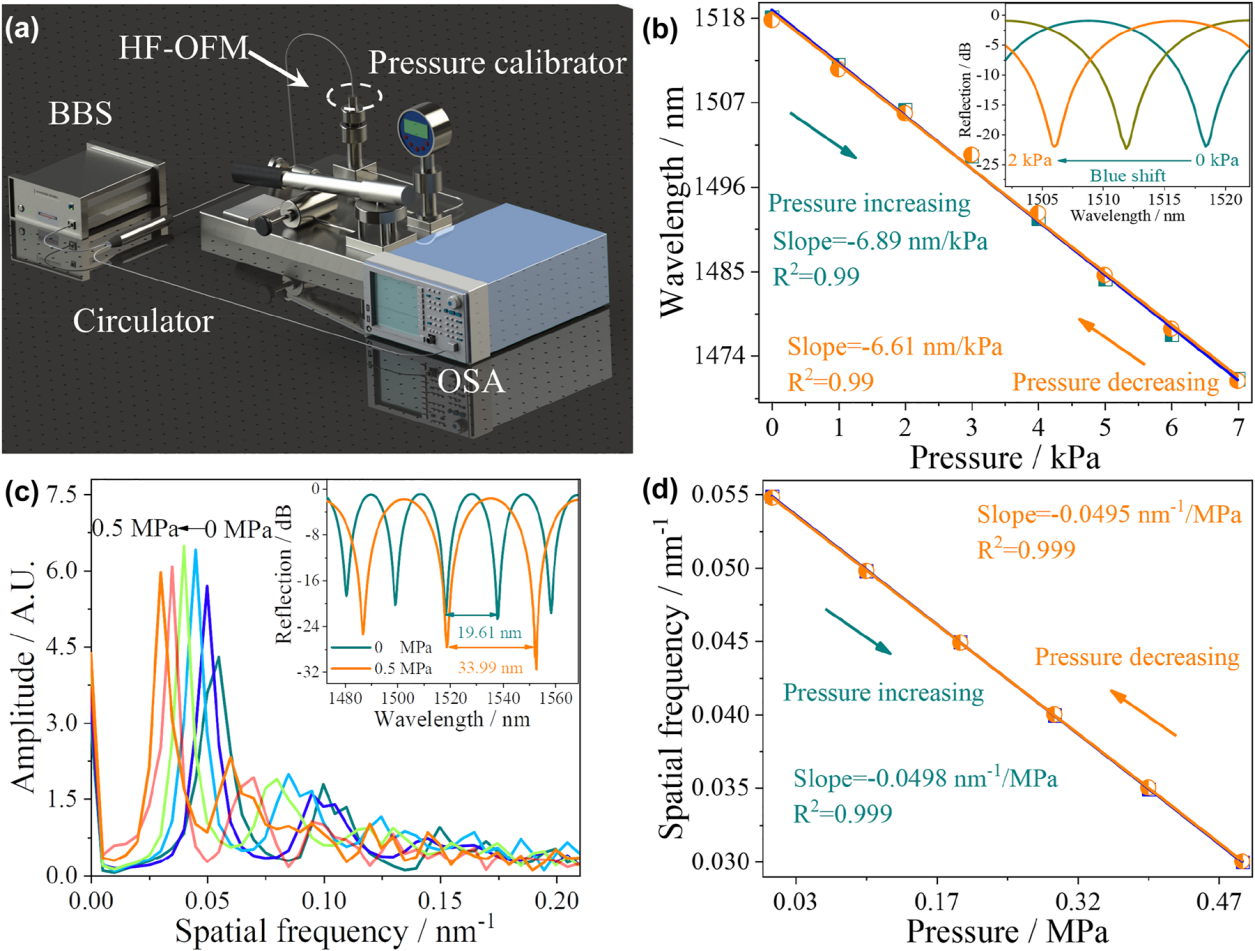
Pressure response characteristics of HF-OFM. (a) Schematic illustration of the experimental setup for the pressure measurement. (b) The wavelength response of the HF-OFM in the pressure range of 0–7 kPa. (c) The spatial frequency spectra obtained at various pressure, (d) and the corresponding frequency response of the HF-OFM in the pressure range of 0–0.5 MPa.

The HF-OFM was sealed in an air pressure calibrator (ALKT702, with the resolution of 1 kPa) to verify the pressure response characteristics. The schematic diagram of the experimental setup is shown in Figure 4(a). The experiment was carried out at room temperature (20 ± 0.5 °C) with the pressure range of 0–7 kPa with an interval of 1 kPa. The corresponding reflection spectra under different pressures are shown in Figure 4(b).

From Figure 4(b), the interference cavity length decreases due to the deformation of the GO film. According to Eq. (1), the shift of resonant wavelength Δ*λ* = *λ*(Δ*n*/*n* + Δ*L*/*L*) is only related to Δ*L*. Thus, the reflection spectrum is blue shifted with the increase of pressure. In order to verify the repeatability and hysteresis of the HF-OFM, the pressure dropped from 7 to 0 kPa, the evolution of the reflection spectrum is opposite of the pressure rise process. By calculation, the sensitivities can reach −6.89 and −6.61 nm/kPa with high linearity (>0.99) in the process of pressure increase/decrease. The pressure response characteristics of the sensor with an interference cavity length of 141.24 μm are shown in Figure S2 with a sensitivity of −2.9 nm/kPa, which proves that the short cavity length can effectively improve the air pressure sensitivity. Thus, the HF-OFM with high pressure sensitivity is sufficient to meet the needs of acoustic signal detection. A good linear relationship between temperature and resonance wavelength with the sensitivity of only −56 pm/°C is demonstrated in Figure S3. Combined with the ∼4.83 nm linear operating region of the structure, this HF-OFM is unaffected by temperature variations when used in a room temperature environment (20 ± 5 °C).

In order to further study the response characteristics of the HF-OFM in high pressure environment, the pressure range was set to 0–0.5 MPa. From the subfigure in Figure 4(c), the FSR of the reflection spectrum is significantly increased by 14.38 nm (from 19.61 nm to 33.99 nm). The reflection spectra were converted to spatial frequency spectra using the fast Fourier transform (FFT) method, as shown in Figure 4(c). It is found that the spatial frequency shifts to the low-frequency direction with the increase of pressure, which means that the length of the interference cavity gradually decreases and the FSR gradually increases. As the pressure decreases, the spatial frequency shifts back to the high-frequency direction. The linear fitting relationships between spatial frequency and pressure are shown in Figure 4(d). The corresponding sensitivities are −0.0498 nm^−1^/MPa and −0.0495 nm^−1^/MPa in the pressure increasing and decreasing, indicating that the HF-OFM can still maintain good linear response characteristics through spatial frequency demodulation in a large pressure environment.

**Figure 5:**
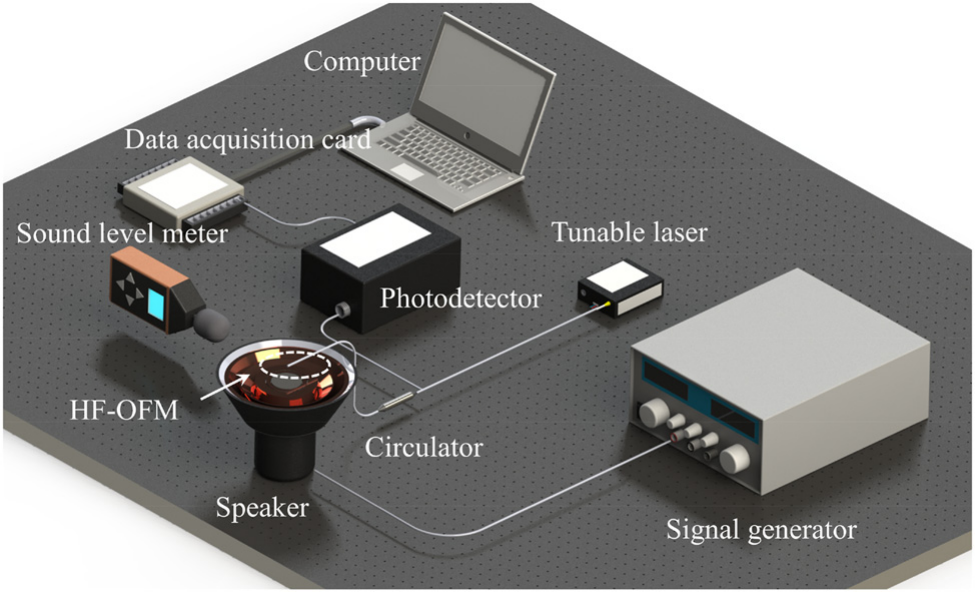
Schematic illustration of the experimental setup for the acoustic signal measurement.

Figure 5 shows the schematic illustration of the experimental setup for the acoustic signal measurement. The optical signal output from the tunable laser (TLS-3000, OPEAK, with the range of 1520–1560 nm) is transmitted through the circulator to the HF-OFM where it is reflected. According to the reflection spectrum of the proposed HF-OFM in Figure 3(k), the center wavelength and power of the tunable laser were set to 1553.2 nm and 7 mW. The reflected light is converted into an electrical signal by a photodetector (KG-PR-200K-A, with the ∼3 dB bandwidth and gain of 200 KHz and 1 × 10^7^ V/W), which is then collected by a data acquisition card (USB-HRF4626, with the sampling rate of 50 KHz for a single channel). When an acoustic signal of a specific frequency is applied to the speaker (XMYX07YM, with the driving voltage of 5 V) through the signal generator (RK1212G, with the range of 0.02–20 kHz), a time domain signal with a fixed voltage amplitude and periodic changes can be observed on the computer. A sound level meter (TA8151, with the resolution of 0.1 dB) was placed near the HF-OFM for calibration.

**Figure 6:**
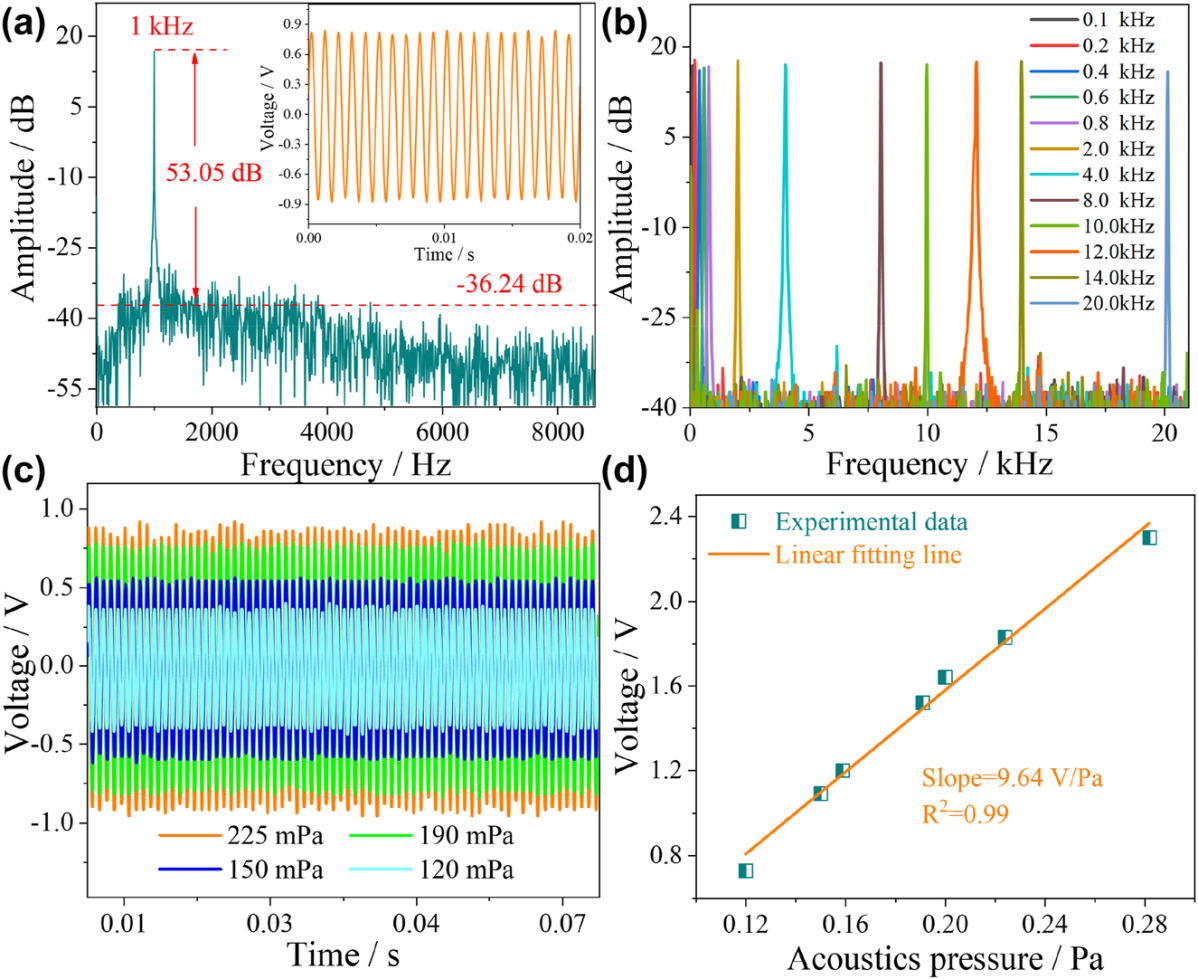
Acoustic frequency response characteristics of HF-OFM in a single-frequency environment. (a) Measured frequency-domain spectrum with a single frequency of 1 kHz. (b) Measured frequency-domain spectra of different frequencies from 0.1 kHz to 20 kHz. (c) The measured time-domain spectra at a single frequency of 1 kHz under different acoustic pressures. (d) The relationship between acoustic pressure and voltage.

The signal generator applied a single-frequency acoustic signal of 1 kHz with an acoustic pressure of 200 mPa, and the corresponding time-domain spectrum is shown in the inset of Figure 6(a). The small voltage amplitude fluctuations in the time-domain spectrum indicate that thermal and equipment noise do not affect signal demodulation. To further analyze the accuracy and MDP of the HF-OFM, the frequency-domain spectrum after FFT of the time-domain spectrum is shown in Figure 6(a). The main peak of the frequency domain spectrum is 1 kHz, which is consistent with the frequency of the input signal. With the 50 Hz resolution bandwidth, the noise floor and SNR are −36.24 dB and 53.05 dB, which are limited by the SNR of the speaker. According to [18], the MDP of the HF-OFM under test is 63.25 μPa/Hz^1/2^. Subsequently, the acoustic signal in the human hearing frequency range of 0.1 kHz–20 kHz was measured, and Figure 6(b) shows the corresponding frequency domain spectra. The experimental results show that the tested frequency is in good agreement with the input acoustic signal, and the average SNR and fluctuation are 53.23 dB and ±0.88 dB. Compared with references [26–28], the response flatness is improved by an order of magnitude, which provides the possibility for the high-fidelity transmission of acoustic signals. In addition, the acoustic pressure of the input acoustic signal can be determined according to the voltage amplitude in the time-domain spectrum. As shown in Figure 6(c), the voltage amplitude increased as the external acoustic pressure increased from 120 mPa to 280 mPa. The linear relationship between the voltage amplitude and the acoustic pressure is shown in Figure 6(d); the acoustic pressure sensitivity is 9.64 V/Pa with the linearity of 0.998. The acoustic pressure sensitivity of the sensor without Au nanocoating is shown in Figure S1(c) and (d), and the sensitivity is only 3.92 V/Pa. In contrast, the acoustic pressure sensitivity of HF-OFM is increased by about 2.5 times, which shows that the Au nanocoating structure has a significant amplification effect on the acoustic pressure sensitivity. The high acoustic pressure sensitivity shows that the HF-OFM has a good detection ability for weak acoustic signals in the audible frequency range of the human ear.

**Figure 7:**
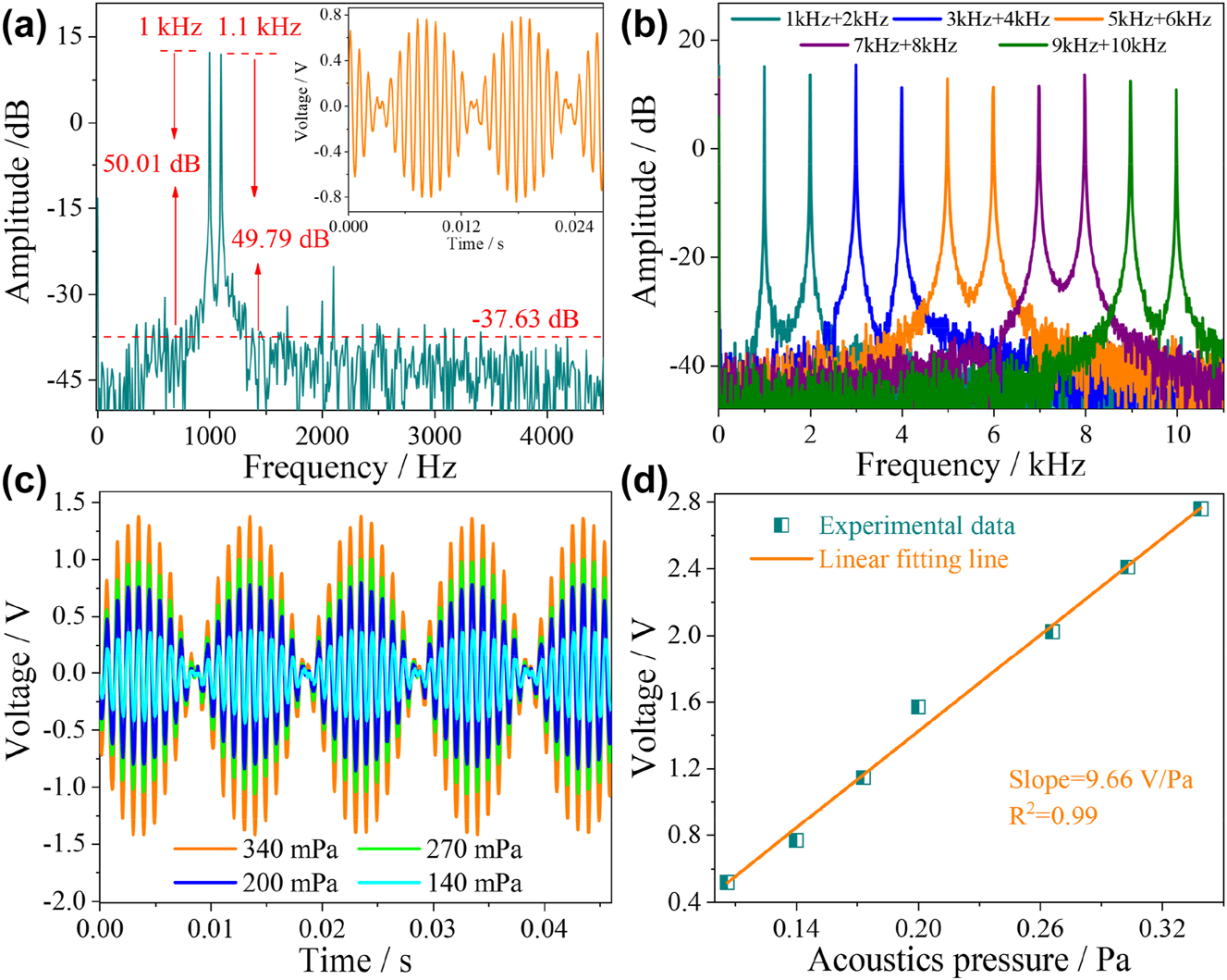
Acoustic frequency response characteristics of HF-OFM in a dual-frequency environment. (a) Measured frequency-domain spectrum with dual frequency of 1 kHz and 1.1 kHz. (b) Measured frequency-domain spectra of different frequencies from 1 kHz to 10 kHz. (c) The measured time-domain spectra at dual frequency of 1 kHz and 1.1 kHz under different acoustic pressures. (d) The relationship between acoustic pressure and voltage.

In acoustic signals detection, the existence of hybrid frequency is inevitable. In order to study the response characteristics of dual frequencies, dual frequency acoustic signal of 1 and 1.1 kHz with an acoustic pressure of 200 mPa was applied to the HF-OFM. The corresponding time-domain and frequency-domain spectra are shown in Figure 7(a), with obvious beat frequency generation in the time-domain spectrum. There are two obvious main peaks at 1 kHz and 1.1 kHz in the frequency domain spectrum, which are consistent with the input acoustic signal. The SNRs are 50.01 dB and 49.79 dB, respectively, and the noise floor is −37.63 dB. The frequency of the generated envelope is 0.1 kHz. In order to directly represent the interaction between multi-frequency acoustic signals, according to Eq. (1), the forced vibration of film can be expressed as

(6) $$\begin{array}{ll} L\left(t\right) & =A\sin\left(\omega_{1}t+\varphi \right)+A\sin\left(\omega_{2}t+\varphi \right)\\ & =2A\left.\left|\cos\left(\frac{\omega_{1}-\omega_{2}}{2}t\right)\right.\right|\sin\left(\frac{\omega_{1}+\omega_{2}}{2}t+\varphi \right) \end{array}$$

where *A* is the amplitude of the acoustic signal, *ω* is the angular frequency, *t* is the time, and *φ* is the initial phase. When two frequencies are close but not equal, a beat phenomenon occurs. Since 0 ≤ |cos(*ω*_1_ − *ω*_2_)*t*/2| ≤ 1, the amplitude of the superimposed simple harmonic is twice that of the original acoustic signal. From Eq. (6), the vibration frequency and envelope frequency of the superimposed simple harmonics with 1 kHz and 1.1 kHz acoustic frequency are |*f*_1_ + *f*_2_|/2 = 1.05 kHz and |*f*_1_ − *f*_2_| = 0.1 kHz, which are consistent with the experimental results.

In the frequency range from 1 kHz to 10 kHz, five groups of dual frequency acoustic signals were tested with the step of 1 kHz, and the corresponding frequency domain spectra are shown in Figure 7(b). The experimental results show that the frequency response of the HF-OFM to the dual frequency acoustic signal still has good accuracy. The average SNR and fluctuation are 50.55 dB and ±2.15 dB. The acoustic pressure response characteristics of the microphone to the dual-frequency acoustic signal are further studied, as shown in Figure 7(c). The voltage amplitude increased as the external acoustic pressure increased from 110 mPa to 340 mPa. The linear relationship between the voltage amplitude and the acoustic pressure is shown in Figure 7(d); the acoustic pressure sensitivity is 9.66 V/Pa with the linearity of 0.99. It is proved that HF-OFM still has high precision detection ability for hybrid frequency acoustic signal.

**Figure 8:**
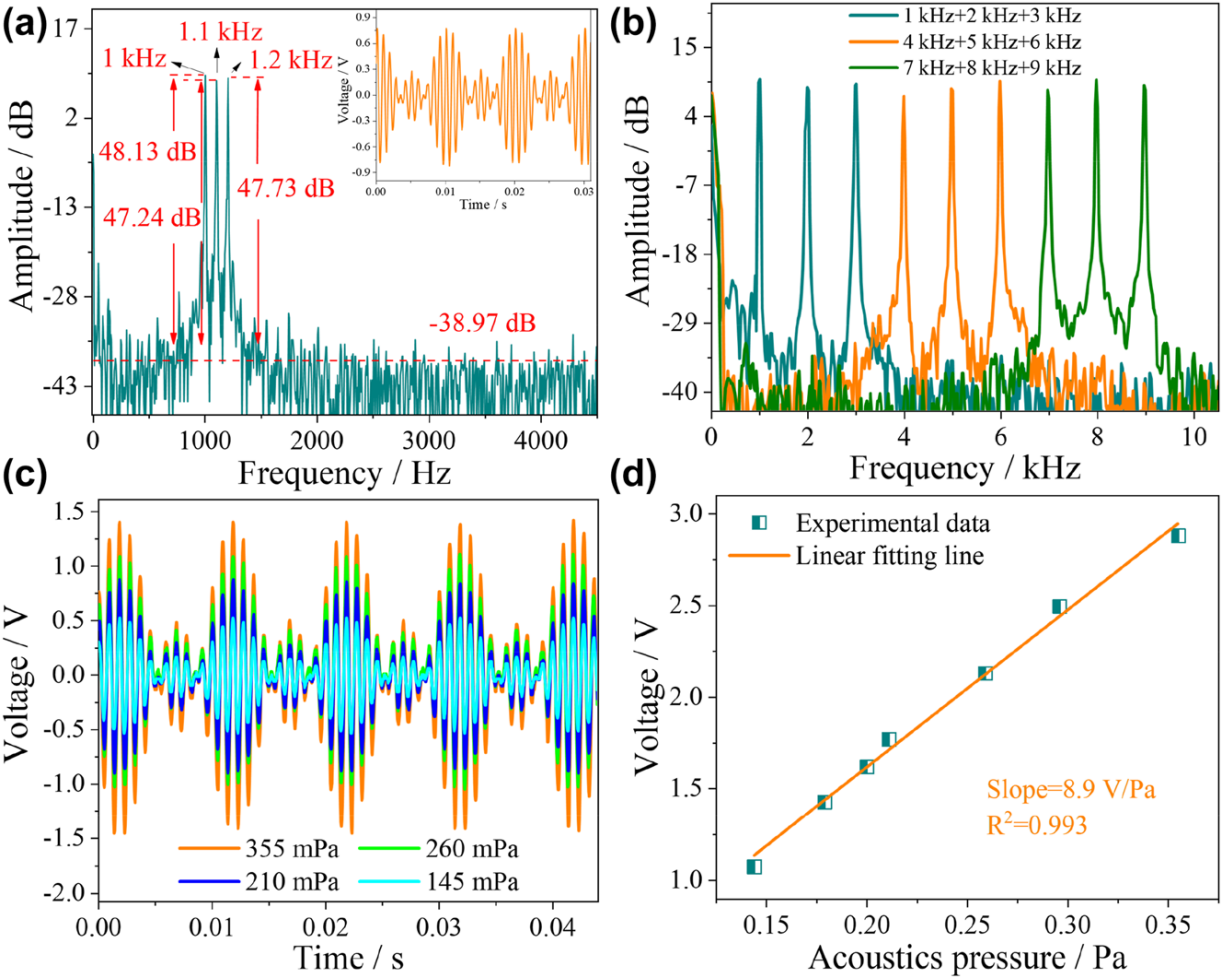
Acoustic frequency response characteristics of HF-OFM in a triple-frequency environment. (a) Measured frequency-domain spectrum with triple frequency of 1 kHz, 1.1 kHz, and 1.2 kHz. (b) Measured frequency-domain spectra of different frequencies from 1 kHz to 9 kHz. (c) The measured time-domain spectra at 1 kHz, 1.1 kHz, and 1.2 kHz under different acoustic pressures. (d) The relationship between acoustic pressure and voltage.

The response characteristics of triple frequencies are further studied. Figure 8(a) shows the time-domain and frequency-domain spectra of triple frequency at 1, 1.1, and 1.2 kHz under the external acoustic pressure of 200 mPa. The frequency main peaks of 1, 1.1, and 1.2 kHz are consistent with the input frequency, and the corresponding SNRs are 48.13, 47.24, and 47.73 dB and noise floor is −38.97 dB. Due to the similar frequencies of the three input acoustic signals, the time-domain spectrum in Figure 8(a) appears a phenomenon similar to beat frequency, with obvious periodic envelope of 0.1 kHz. The voltage amplitude is a linear superposition between the three frequencies and is, therefore, the same as the amplitude of the single and dual frequencies under the same input power condition, which are both around 1.6 V. In the frequency range from 1 kHz to 9 kHz, three groups of triple-frequency acoustic signals were tested with the step of 1 kHz, and the corresponding frequency domain spectra are shown in Figure 8(b). The experimental results show that the frequency response of the HF-OFM to the triple-frequency acoustic signal still has good accuracy, and the average SNR and fluctuation are 47.59 dB as and ±1.22 dB. According to the law of energy conservation, the SNRs of the main frequency peaks in the dual-frequency and triple-frequency inputs are theoretically reduced by 1/2 (3 dB) and 1/3 (4.77 dB), respectively, which is consistent with the test results in our experiments. Among them, the small errors mainly come from the other signal noises brought by the multi-frequency input [33]. The experimental results also demonstrate that the proposed preparation method can effectively improve the stability of the GO film and reduce the loss of the input signal at the GO film. As the acoustic pressure increases, the trend of the voltage amplitude of the triple-frequency acoustic signal is consistent with that of the single-frequency and dual-frequency signals, as shown in Figure 8(c). The linear relationship between the voltage amplitude and the acoustic pressure is shown in Figure 8(d); the acoustic pressure sensitivity is 8.9 V/Pa with the linearity of 0.993. For single-frequency and multi-frequency acoustic signals detection, the acoustic pressure sensitivity at the same frequency is basically the same. With its good detection ability for hybrid-frequency signals, the HF-OFM has broad application prospects in the field of real-time communication.

**Figure 9:**
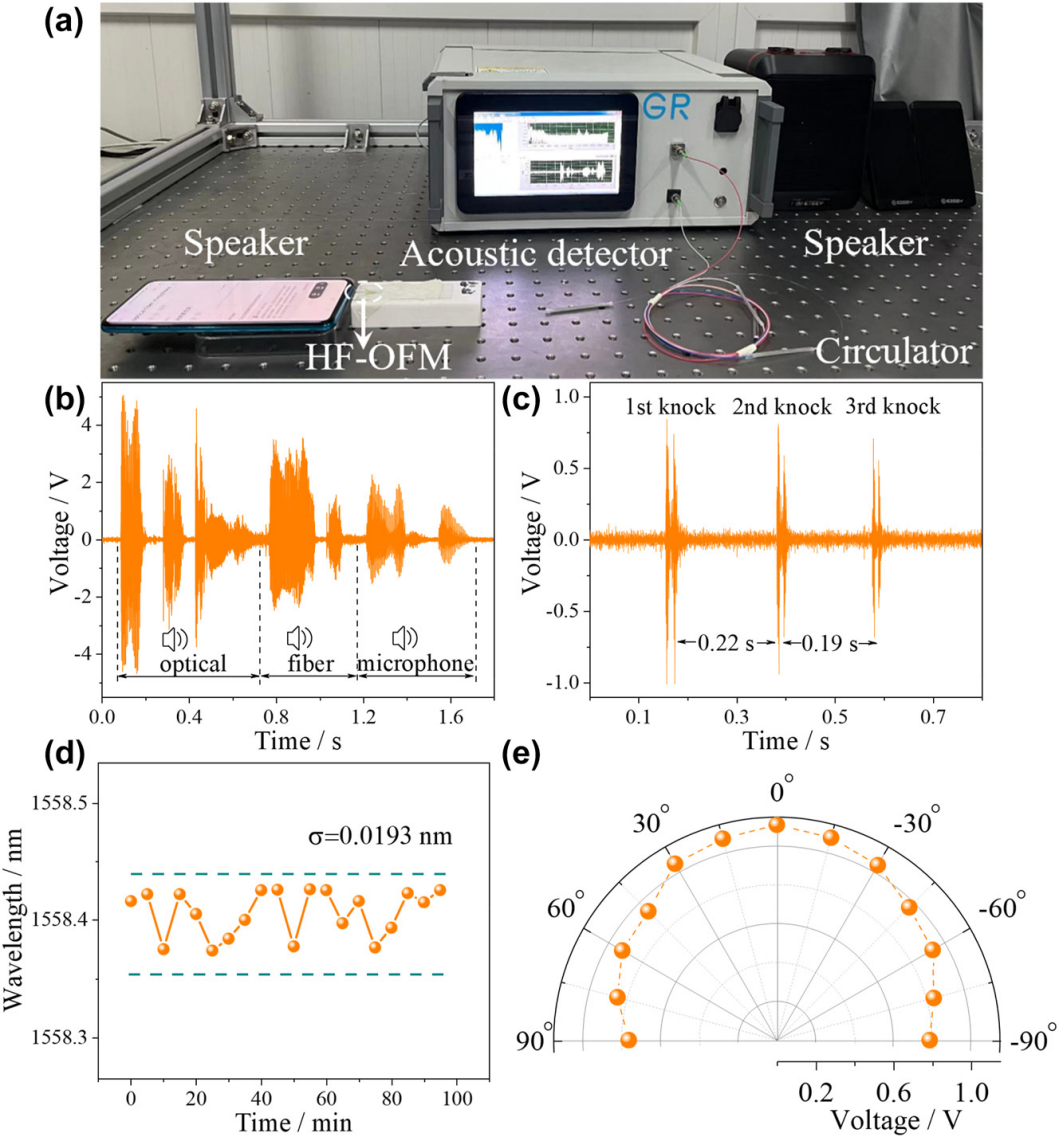
High fidelity verification, stability testing and omnidirectionality testing of HF-OFM. (a) Real-time testing of acoustic signal transmission. The measured time-domain spectra of (b) the “optical fiber microphone” and (c) the percussion table. (d) Stability test results and (e) directional response of the HF-OFM.

In order to further explore the application potential of HF-OFM in the field of real-time communication, a filtering module has been added to the experimental setup in Figure 9 to filter the digital signal from the acquisition card, retaining only the frequency band audible to the human ear. To facilitate the measurement, demodulation, and playback of the acoustic signal, an optical fiber acoustic detector was prepared as shown in Figure 9(a). The HF-OFM was connected to the optical fiber acoustic detector using a circulator and an external speaker outputs the acoustic signal received by the HF-OFM. As shown in Figure 9(a), the mobile phone acting as a speaker plays a piece of “optical fiber microphone” audio, corresponding to the time-domain spectrum shown in Figure 9(b). Due to its high SNR, the HF-OFM provides an accurate recording of the loudness, tone, and timbre of the input audio and is capable of outputting a lossless audio signal in real time, as shown in Movie S1 (Supporting Information). It also provides lossless real-time transmission of the sound of a desk being struck several times in rapid succession, as shown in Figure 9(c) and Movie S2 (Supporting Information), at a frequency of ∼0.2 s. With its high accuracy, fast response time, and lossless real-time transmission, the proposed HF-OFM has great potential for applications such as listening and optical telephony.

**Movie S1:** 

**Movie S2:** 

The directivity measurement is necessary for characterizing an optical fiber microphone. The HF-OFM was fixed on a rotary stage 5 cm from the sound source, defining a position of 0° at the microphone directly facing the speaker. The angular test range was from −90° to 90°, and the amplitude of the time-domain spectrum of the acoustic signal at 1 kHz was recorded at 15° intervals, and the experimental results are shown in Figure 9(e). As the angle increases, the amplitude of the time domain spectrum tends to decrease slightly. The acoustic amplitude is highest at 0° and decreases by only 0.31 V in the range of −90° to 90°. Thus, the HF-OFM developed in this work has good omnidirectionality.

To further verify the stability of this HF-OFM, the outside temperature and pressure were maintained at 20 °C and standard atmospheric pressure. Spectra were recorded 20 times continuously with an interval of 5 min. The results are shown in Figure 9(d); the average and standard deviation *σ* of 1558.406 nm and 0.0193 nm, respectively, indicating good stability of the microphone. Combined with the low temperature crosstalk, the *Q* point of the proposed microphone is relatively stable. From Table S1, the proposed HF-OFM achieves high acoustic pressure sensitivity in the frequency range of 0.1–20 kHz compared to the currently reported optical fiber acoustic sensors. And the frequency response is flat, and the fluctuation is only 1.76 dB, which has been improved by nearly an order of magnitude. Therefore, HF-OFM enables real-time transport of high-fidelity audio.

## 4 Conclusions

In summary, a sensitivity-enhanced and flat response HF-OFM was theoretically and experimentally demonstrated by the means of intensity demodulation–based photoelectric conversion method. The F–P cavity is composed of two kinds of nanofilms, namely GO film and Au nanocoating by mechanical stretching method and magnetron sputtering method. To improve the acoustic pressure sensitivity, the GO film was optimized by numerical analysis. The pressure sensitivity is up to −6.89 nm/kPa and can withstand a maximum pressure of 0.5 MPa with the resonance frequency of 315.87 kHz. The acoustic pressure sensitivity is further amplified by the design of the 3 nm Au nanocoating and the 58.95 µm cavity length. Due to the high sensitivity (9.64 V/Pa) and flat response (<2 dB variation) of the GO film, the single, dual, and triple frequencies SNRs are 53.05 dB, 50.01/49.79 dB, and 48.13/47.24/47.73 dB at 1/1.1/1.2 kHz, with the MDP of 63.25 μPa/Hz^1/2^. In addition, the HF-OFM has a good acoustic pressure response in the angle range of −90° to 90°. What’s more, the HF-OFM has been experimentally proven to be capable of real-time transmission of high-fidelity audio signals by virtue of its hybrid-frequency detection capability. With its high sensitivity, omnidirectionality, small size, and immunity to electromagnetic interference, the HF-OFM has great potential for real-time communication applications.

## Supplementary Material

Supplementary Material Details
