# Supplementary material for: High-fidelity optical fiber microphone based on graphene oxide and Au nanocoating
Source: Nanophotonics. 2023 Sep 20;12(19):3707–19. doi: 10.1515/nanoph-2023-0209 (PMC11636454; doi:10.1515/nanoph-2023-0209)
Supplement: Supplementary file 5 — Supplementary Material Details [file j_nanoph-2023-0209_suppl_001.pdf]

Supplementary Information for

High-fidelity optical fiber microphone based on graphene oxide and Au nanocoating

Liangtao Hou[1], Yan Li[1,*], Libin Sun[1], Chao Liu[2], Yichao Zheng[1], Yi Liu[1] and Shiliang Qu[3]

[1]Department of Optoelectronics Science, Harbin Institute of Technology, Weihai 264209, China

[2]Sino-German Joint Research Center of Advanced Materials, School of Materials Science and Engineering, East China University of Science and Technology, Shanghai 200237, China

[3]School of Physics, Harbin Institute of Technology, Harbin 150001, China

*Corresponding author. Email: liy@hit.edu.cn.

This file includes:

Figs. S1, S2 and S3

Table S1

Supplementary descriptions 1-5

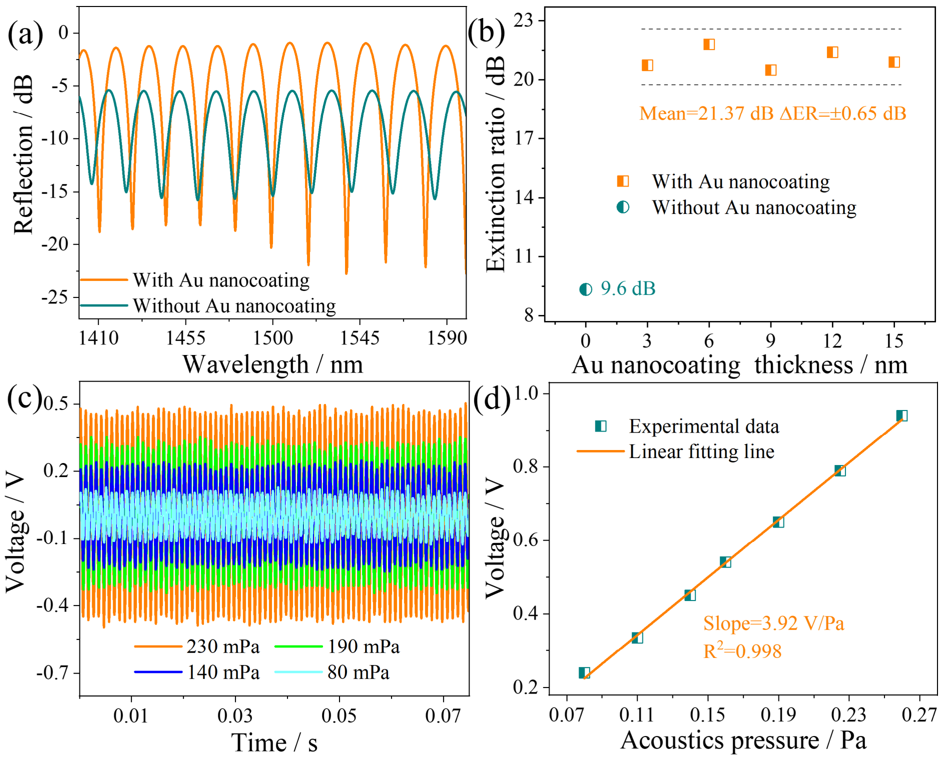

Fig. S1: (a) HF-OFM reflection spectra with and without Au nanocoating. (b) Reflection spectra of different thickness of Au nanocoating. (c) The measured time-domain spectra at a single frequency of 1 kHz under different acoustic pressures. (d) The relationship between acoustic pressure and voltage.

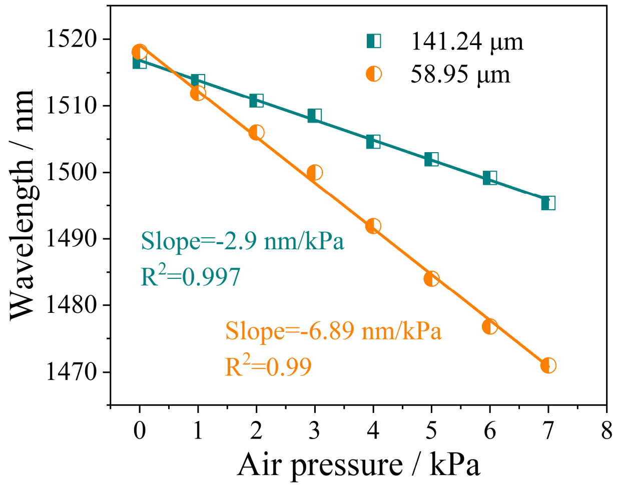

Fig. S2: Linear relationship between wavelength and air pressure at interference cavity lengths of 58.95 μm and 141.24 μm.

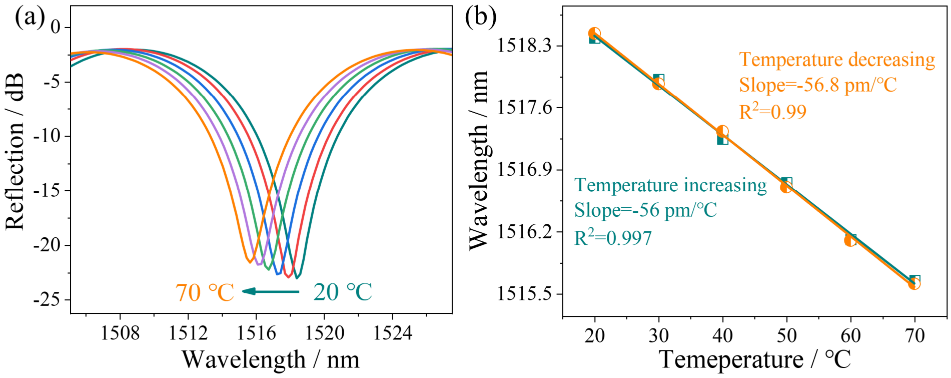

Fig. S3: (a) The reflection spectrum with varied temperature and (b) the corresponding wavelength response.

Table S1. Performance comparison of different optical fiber acoustic sensors

Film material	Sensitivity	Frequency range	MDP	Flat response	Multi-frequency detection capability	Audio transmission capability
Chitosan[16]	52.6mV/kPa @180kHz	100-800kHz	2.37mPa/Hz$^{1/2}$ @180kHz	19.5dB	No	No
Glass[19]	755mV/Pa @0.5kHz	32-800Hz	126μPa/Hz$^{1/2}$ @500Hz		No	No
Al[14]	34.8 nm/kPa @1.4kHz		0.26Pa/Hz$^{1/2}$ @1.4kHz		No	No
Biophotonic structure[28]	13.9mV/Pa @1kHz	0.2-4.5kHz		~10dB	Yes	No
GO[26]	0.102V/Pa @20kHz	4-20kHz	28.74μPa/Hz$^{1/2}$ @13kHz	~20dB	Yes	No
GO[27]	~700mV/Pa @10 kHz	0.1-20kHz	17.3μPa/Hz$^{1/2}$ @2kHz	~13dB	No	No
This work	9.64V/Pa @1kHz	0.1-20kHz	63.25μPa/Hz$^{1/2}$ @1kHz	1.76dB	Yes	Yes

Supplementary descriptions 1 (Figs. S1(a)-(d))

The reflection spectra with and without the Au nanocoating for an interference cavity length of about 60 μm are shown in Fig. S1(a). It is evident that the free spectral ranges of the reflection spectra are equal. Compared with the reflection spectrum without Au nanocoating, the extinction ratio (ER) of the reflection spectrum with Au nanocoating increased by 9.62 dB. Subsequently, the thickness of the Au nanocoating was changed by controlling the time of magnetron sputtering, and the thickness of the prepared Au nanocoating ranged from 0 to 15 nm with a step size of 3 nm. The ERs of the reflection spectra of the Au nanocoating with different thicknesses are shown in Fig. S1(b). The Au nanocoating effectively improves the ER of the reflection spectrum. And the ER does not change significantly with the increase of Au nanocoating thickness. In the thickness range of 3-15 nm, the average value of the ER is 21.37 dB, and the fluctuation is only ±0.65 dB. Therefore, in terms of the low cost of the microphone, the thickness of the Au nanocoating was chosen to be 3 nm.

To further study the acoustic pressure sensitivity of the Au-free nanocoating structure, the voltage amplitude increased as the external acoustic pressure increased from 80 mPa to 260 mPa, as shown in Fig. S1(c). The linear relationship between the voltage amplitude and the acoustic pressure is shown in Fig. S1(d), the acoustic pressure sensitivity is 3.92 V/Pa with the linearity of 0.998.

Supplementary descriptions 2 (Figs. S2)

The sensors with cavity length of 58.95 μm and 141.24 μm were sealed in pressure calibrators to verify the pressure response characteristics. The experiment were carried out at room temperature (20±0.5 °C) with the pressure range of 0 to 7 kPa with an interval of 1 kPa. The corresponding reflection spectra under different pressures are shown in Fig. S2. The sensitivities are -6.89 nm/kPa and -2.9 nm/kPa, respectively, which are consistent with the theoretical analysis. It was verified that the short cavity length effectively improved the air pressure sensitivity.

Supplementary descriptions 3 (Figs. S3)

The HF-OFM was then fixed in a drying oven (LICHEN, China) heated from 20 to 70 °C with a step size of 10. It can be obviously observed that with the increase of temperature, UV curable adhesive drives the optical fiber to move due to thermal expansion effect, resulting in a decrease in interference cavity length. Therefore, the reflected spectrum undergoes a significant blue shift. In order to verify the temperature stability, temperature rise and temperature drop tests were carried out. The linear fitting relationship between the corresponding wavelength and temperature is shown in Fig. 9(d). The temperature sensitivities are -56 pm/°C and -56.8 pm /°C, with high linearity (> 0.99) and good stability.

Supplementary descriptions 4 (Movie S1)

The playback of the acoustic signal in the optical fiber acoustic detector is set to a delay of 2 s. When the mobile phone plays the audio of the "optical fiber microphone", the HF-OFM receives the input acoustic signal. After 2 s, the audio of the "optical fiber microphone" is played back with high fidelity.

Supplementary descriptions 5 (Movie S2)

The playback of the acoustic signal in the optical fiber acoustic detector is set to a delay of 2 s. When the volunteer tapped on the table three times, the HF-OFM receives the vibration signal from the desktop. After 2 s, the vibration signal is played back with high fidelity.
